# Which factor explains the life‐history of *Xanthium strumarium* L., an aggressive alien invasive plant species, along its altitudinal gradient?

**DOI:** 10.1002/pld3.375

**Published:** 2022-01-09

**Authors:** Rafi Ullah, Nasrullah Khan, Kishwar Ali

**Affiliations:** ^1^ Department of Botany University of Malakand Chakdara Pakistan; ^2^ School of General Education College of the North Atlantic – Qatar Doha Qatar

**Keywords:** elevation gradient, functional traits, invasive success, Pakistan, phenological shift, reproductive performance

## Abstract

Invasive biology acknowledges the concept of better performance by invasive plants in the introduced range. *Xanthium strumarium* L. is one of the successful invasive species in Khyber Pakhtunkhwa, Pakistan. The phenological pattern, vegetative and reproductive traits plasticity analysis of the species was explored to explain the invasive success across the altitudinal gradient in the current invaded habitats. Phenological patterns and timing (seedling, vegetative growth, flowering and fruiting, drying, and seed bank) were observed during a full year for two seasons. We also examine plant functional traits at altitudes of 500, 1000, and 1500 m a.s.l. to assess traits and biomass variations. The *X. strumarium* exhibits late vegetative and reproductive phenology at higher altitudes, enabling them to occupy an empty niche and benefit from decreased competition for resource acquisition. The lower altitude plants show a higher growth rate (stem size increase, number of leaves, and leaf area) due to the higher nutrient availability. Higher altitude plants have the highest reproductive biomass and biomass ratio revealing plant abilities to be reproductively adapted in the higher altitudes. Among climatic variables, mean yearly temperature, mean annual yearly humidity, and mean day length in hours, while in soil variables, organic matter and nitrogen percentage significantly affect the phenological and morphological stages. Therefore, we conclude that *X. strumarium* can invade higher altitudes with a shift in its phenological and morphological changes making the invasion process successful.

## 1 INTRODUCTION

In recent decades, there has been growing research interests in understanding the causes and consequences of biological invasions (Castro‐Díez et al., 2014; Pyšek et al., 2012; Van Kleunen, Weber, et al., 2010; Vilà et al., 2011), owing to the social and economic ramifications of some invasion processes (Andreu et al., 2009; Pimentel et al., 2005). Numerous studies have connected functional characteristics to invasive plant success, such as rapid growth rates, low tissue cost production, capacity to reprocess, low seed size, large number of seed production, extended periods of flowering or N‐fixation (Daehler, 2003; Lloret et al., 2005; Pyšek & Richardson, 2008; Van Kleunen, Dawson, et al., 2010). Many other ecosystem characteristics that increase the invasion rate include resource availability and variations, disturbance frequency, empty niches, biogeographic isolation, and heterogeneity of the habitats (Catford et al., 2009; Dawson et al., 2009; Melbourne et al., 2007). However, most researchers concur that plant features and ecosystem features linked to invasions are context‐dependent (Daehler, 2003; Dawson et al., 2009). Therefore, the functional difference between invasive species and predominant native vegetation has been proposed to influence the likelihood of success and the extent of the effect (Castro‐Díez et al., 2014; Fargione et al., 2003).

The climatic characteristics of any area, particularly the temperature, relative humidity, and day length, acts as an abiotic filter for newly invading aliens (Begum et al., 2021; Richardson et al., 2000). The effectiveness of this filter is predicted by how close climate conditions coincide in the native and invaded areas of the species (Thuiller et al., 2005). When the climate in the new range is comparable to the native range, the phenology of alien species will not vary. It would generate seeds, propagules, vegetative growth, and bloom similarly to its native ranges. On the other hand, in the opposite scenario, the success of alien species is controlled by a combination of the magnitude of climate variations, the inherent capacity of species to flower despise, and the intrinsic ability of species to flower despise (Dudley, 2004). The severity of the climatic filters is linked to changes in functional characteristics such as seedlings, vegetative development, blooming, seed production, and seed banks in the invaded area. If such filters do not restrict invaders, the phenology of their native range will be maintained in invaded regions (Godoy et al., 2009).

Phenotypic plasticity is a unique phenotype displayable under various environmental conditions for a given characteristic (Pfennigwerth et al., 2017). The survival of a species in new habitats and severe circumstances is based on plasticity (Hendry, 2016; Nicotra et al., 2010). Plasticity study is critical to comprehend short‐term adaptive responses (Pfennigwerth et al., 2017). According to Gratani (2014), plant functional traits (PFTs) with climate change reaction versatility are essential in struggling with invasive and indigenous species. Plasticity has previously been documented in morphological characteristics such as plant height, internode length, and shoot numbers, but the current trends are biomass allocation and relative growth, and rate of assimilations (Masarovičová et al., 2015). To adapt to changing environmental circumstances, exotic plants resort to various morphological and physiological changes (Ali et al., 2014; Horton & Neufeld, 1998; Khan et al., 2020, 2021). Studies have shown that the variables of biomass allocation and reproductive time are strong predictors of plant fitness. Changing resource allocation for alien species results in “dispersal ability, dispersion, resistance, and speciation,” according to Claridge and Franklin (2002). Concerning life‐history strategies, the overwhelming bulk of invasion studies have been performed in areas between 0 and 500 m (a.s.l.) (Mostert et al., 2017; Najberek et al., 2020), but the hypothesis that subtropical invaders cannot reach higher elevations because of lower anthropogenic influence and harsh environmental conditions have only been investigated in areas above 500 m (a.s.l.) (McAlpine et al., 2015; Najberek et al., 2020).

Climate change and globalization have enabled invasive species to spread and become more common in high‐altitudinal areas (Shrestha & Shrestha, 2019). Because an increase in altitude changes the microclimate that directly affect invasion process (Wilson et al., 1992). Several other meteorological conditions may accompany greater altitudes (Pauchard et al., 2009). These factors may include a reduced supply of resources, shorter growing seasons, lower microbial activity, and a smaller human population density (Ahmad et al., 2020; Ali, 2017). An altitude‐based change to a plant species' functional characteristics may significantly affect the species' attributes (Ali et al., 2014; Trunschke & Stöcklin, 2017). Pauchard et al. (2009) argue that one of the forces leading to alien plant invasion is the pre‐adaptation techniques used by invaders, such as the selection of stress‐tolerant genotypes and exposing species to progressively colder environmental conditions. As reported by Van Kleunen et al. (2011), before its spread, a species increases its biomass production in its native area, preparing it for future spread. Watermann et al. (2020) state that PFT's assist in the survival and expansion of alien species across a wide range of climate and edaphic conditions. Since both plasticity and adaptability variation in variations are frequent occurrences among invading plants, the impacts of subtropical invasive species on native plant populations are poorly understood (Ahmad et al., 2002; Ali et al., 2018; Colautti et al., 2017). *Xanthium strumarium* L. (Asteraceae) is among the world's most prevalent subtropical invaders (Cowie et al., 2019) and has quickly invaded high altitude areas: and were therefore chosen as a model plant for the present research. The aim of the study is to explain the invasive success of *X. strumarium* by evaluating the phenological adaptation in the prevailing climatic condition and phenotypic plasticity across the altitudinal gradient by hypothesizing that (1) the exotic species differ in phenology along the altitudinal gradient (2) PFT,s varies significantly along the altitude in this invasive species (3) successful invaders are expected to be pre‐adapted to more stable higher elevation by less changes in reproductive biomass plasticity.

## 2 MATERIALS AND METHODS

### 2.1 Study system and species

**FIGURE 1:**
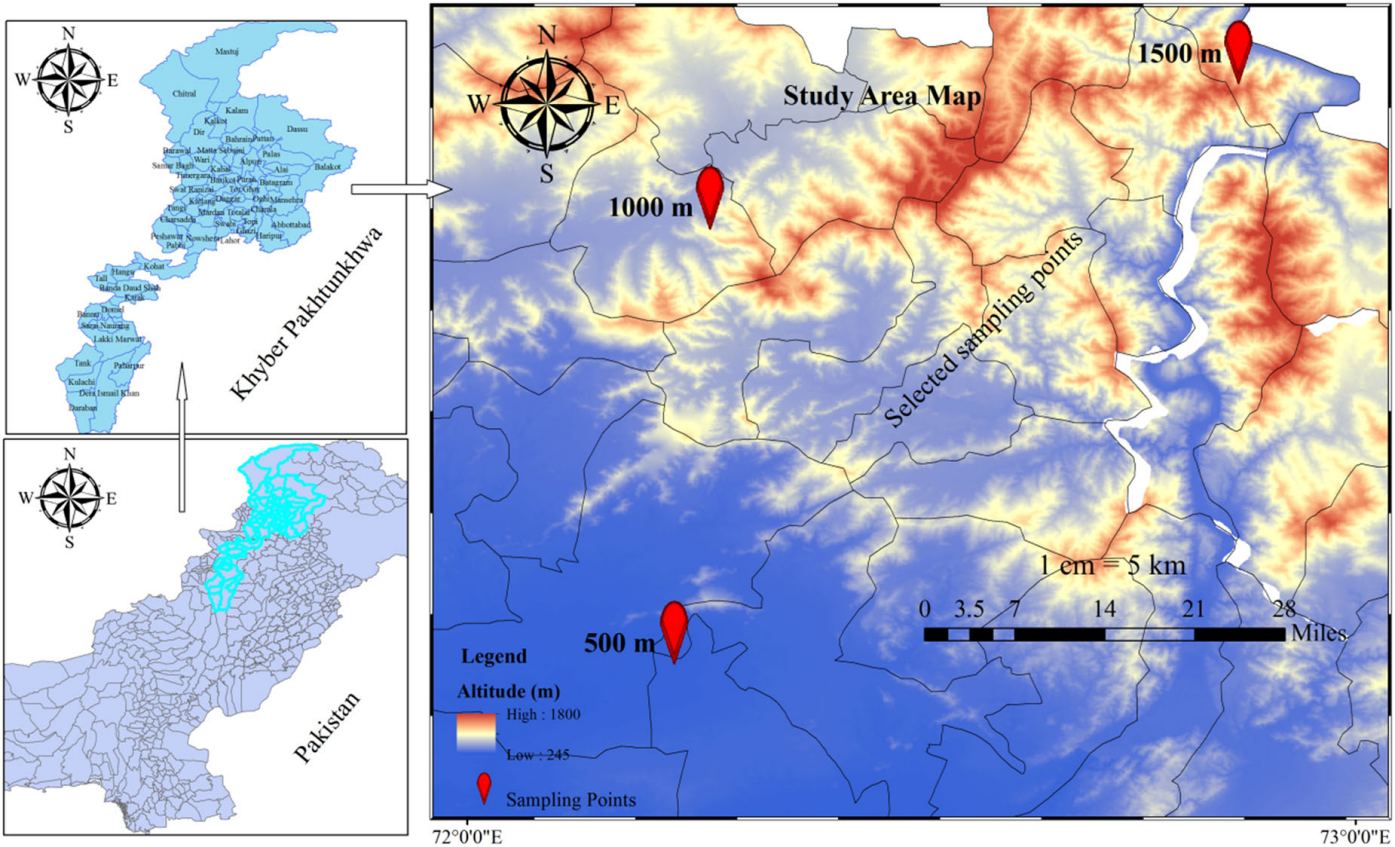
Map of the study area in Pakistan showing sampling points along altitudinal gradient in study area

This research was performed by sampling from lowland up to 1500 m altitude above sea level (m, a.s.l), a public non‐protected region where authorized access is not required. The height of the territory was divided into three different zones, 500, 1000, and 1500 m, a.s.l. Human populations and agricultural activities mostly affect the lower altitudes and have an abundance of alien species (Shah & Sharma, 2015). In contrast, the high‐altitude area is mainly dominated by the subtropical pine and deciduous forests, and little land is utilized for agriculture, wood products, and pastures (Shah & Sharma, 2015). We performed some initial surveys to identify locations where *X. strumarium* is the most prevalent dominant species across the plains and hills in Khyber Pakhtunkhwa, Pakistan, where observational plots (50 m × 50 m) were established along the highway at 500 m, 1000 m, and 1500 m (a.s.l.) in altitude (Figure 1). The quadrat sampling technique was used to assess the plant community structure and composition in each plot (Bellhouse, 2005). Environmental conditions and plant structure throughout this region are regarded as diverse, extending across 74,506 km^2^ with a variety of terrain consisting of plains, undulating areas, and mountainous regions all over the hilly parts. The hilly areas have cold winter and cool summer compared to the plain areas where gradual increase occurs. The province's major contributor to gross domestic product is agriculture, primarily dependent on precipitation and rarely irrigated fields. The area's principal crops are maize, wheat, rice, sugar cane, and tobacco (Pakistan Bureau of Statistics, 2017). Thirty‐two‐year precipitation, temperature and relative humidity data show uniform periodicity with a slight variation of temperature on the plain, that is, .4°C per year (Ali et al., 2018).

*The X. strumarium* L. (Common cocklebur), an annual, seed germinated species of family Asteraceae and widely acknowledged as an alien invasive weed of cotton, groundnut, corn, and soybean crops, is native to North America and Argentina (Hare, 1980; Nel et al., 2004; Venodha, 2016). The plant can tolerate a broad range of temperature invading ruderal and marshlands and is considered one of the drought‐resistant invasive plant species (Arifa et al., 2014). Biological and taxonomical characteristics and their impacts on crops and the economy were described in detail by Weaver and Lechowicz (1983) and Nigussie et al. (2017). The plant was reported as a weed in Pakistan by Baloch et al. (1968) and has rapidly spread in Northern areas over the last two decades along road, urban and rural areas affecting native plants and crops (Hussain et al., 2014). The rapid spread of species in borderline areas of Pakistan‐Afghanistan is strengthening the hypothesis that invasive plants are transported through inter‐continental traffic (Li et al., 2010). The *X. strumarium* has allergenic pollen that causes skin allergies in individuals in contact with glandular hairs and contributes to decreasing crops' yield and losses (King, 1967). For example, a 5% yield loss in cotton was reported in Mississippi (Snipes et al., 1982), and 6%–27% in North Carolina (Byrd & Coble, 1991), with these yield losses occurring 2–10 weeks after invasive species emergence (Snipes et al., 1982). Similarly, a reduction in yield is directly related to plant density. For example, groundnut yield is affected by 31%–39% in a density of .5 plants/m^2^ and may reach ~88% if the density increases to 4 plants/m^2^ (Royal et al., 1997)*. The effects of X. strumarium infestation on maize are less severe than on cotton, soybeans, and groundnut, with a 10% yield decrease occurring at 1 plant/m* ^*2*^ *and reaching 27% (4.7 plants/m* ^*2*^ *), whereas a 5%–50% reduction in yield of snap beans were recorded with a density of .5–8 plants/m* ^*2*^ (Beckett et al., 1988; Neary & Majek, 1990). Nonetheless, crop yield in horticulture is reduced (Weaver & Lechowicz, 1983). Plant species invasion reduces fodder plant yield, which reduces the number of livestock products, particularly for sheep, cattle, and pigs (Kamboj & Saluja, 2010; Seifu et al., 2017).

### 2.2 Fieldwork design

Patches of *X. strumarium* are conspicuously evident along the roadside due to its transfer across Afghanistan by refugees with their sheep and goats during the Afghan nomad migration (Hashim & Marwat, 2002), and it is now a significant component of the ecosystem. We chose 300 healthy plants per elevation group in February 2018, before any signs of bud break, to conduct phenological observations until the life cycle's completion. We monitored phenology from November 1, 2018 until October 20, 2020, when *X. strumarium* fruit dispersion was completed. Every 1–3 weeks, depending on the plant's activity, we assessed if the following phenophases were active or not: Seedling emergence, vegetative growth, flowering and fruit set, fruit dispersion, and seed bank. When phenophases could be seen with the naked eye on at least 5% of the crown, they were considered active. Climatic conditions, i.e., mean monthly temperature (MMT), annual and monthly humidity (AMH), and day length in hours (DLH) were recorded for 2 years in each phenophases.

Plots (50 × 50 m) were created 50 m from the road at altitudes of 500, 1000, and 1500 m. a.s.l. in previously reported *X. strumarium*‐dominated locations in the Northern plains and hilly regions of KPK. The vegetation structure and composition of each plot were assessed using the quadrat method (Bellhouse, 2005). The observational plots were divided into three (10 m × 10 m) plots, each of which was dissected into three (5 m × 5 m) sub‐plots for vegetation recording. Selected sampled sites were found in deforested regions or wastelands. Vegetative trait analyses were carried out by digging up 105 healthy and mature individuals of *X. strumarium* from each study site (1 plant collected from the center of each 5 m × 5 m quadrat; 500, 1000 , 1500 m) and transporting them to the laboratory in damp towels. Similarly, in terms of functional reproductive traits, capitula and fruit count/plant with dry biomass were performed manually at each site. To assess trait adaptability in PFT's, 11 vegetative and four reproductive characteristics were assessed by following Perez‐Harguindeguy et al. (2016). Above‐ground plant height/plant (AGPH/P; cm), root length/plant (RL/P; cm), crown cover/plant (CC/P; cm), number of leaves/plant (NL/P), leaf length/leaf (LL/L; cm), leaf width/leaf (LW/L; cm), leaf area/leaf (LA/L; cm^2^), leaf dry biomass/plant (LDBM/P; g), above‐ground dry biomass/plant (ADBM/P; g), below‐ground dry biomass/plant (BDBM/P; g) and total dry biomass/plant (TDBM/P; g) were the vegetative traits included in the analysis. Reproductive traits studied included: number of capitula/plant (NC/P), number of fruits/plant (NF/P), Inflorescence dry biomass/plant (IDBM/P; g), Fruit dry biomass/plant (FDBM/P; g), number of capitula to number of fruit (NC/NF) and inflorescence dry biomass to fruit dry biomass (IDBM/FDBM). The individual plants collected were measured for their AGPH/P, RL/P, CC/P, LL/L, and LW/L by using a measuring tape. The NL/P, NC/P, and NF/P were counted manually at the study sites. Following Sharma et al., 2017, various functional parts of the sampled plants, namely NL, above and below‐ground biomasses, NC and NF, were oven‐dried for 72 hours at a temperature of 60°C and then weighted by electronic balances to determine LDBM/P, ADBM/P, BDBM/PP, IDBM/P, and FDBM/P with a precision of = .0101 g. The NC/P and NF/P of the sampled plants were dried. In the AGDB/P measurement FDBM/P was omitted. For the computation of biomass allotment, dry matter content (g) was utilized following Kaur et al. (2019). AGDB/P, BDBM/P, IDBM/P, and FDBM/P were included in the percentage of the biomass allocation.

### 2.3 Soil analysis

To assess the soil conditions/properties, soil samples were taken from the center of each 5 m × 5 m quadrat between 5 and 15 cm depth from all three kinds of sites (500, 1000, and 1500 m) and thoroughly mixed to produce three duplicates (*n* = 3) per site. After air‐drying and screening, the soil was analyzed for textural classes using the Huluka and Miller (2014) hydrometric technique. Similarly, soil pH (solution 1:2 w/v), total nitrogen (N %) was determined using the Kjeldahl method, total phosphorous (P mg/kg) was quantified using the ammonium vanadate molybdate method, total potassium (K mg/kg) was extracted using the ammonium acetate extraction method, and organic carbon (OC%) was determined by using the protocol adopted by Tandon (1993). Electrical conductivity (EC: μS/cm) was also determined for each site's soil sample by calibrating (using .01 KCl solution) a digital conductometer (Model CC601 Century) according to the procedure adopted by Tandon (1993). Furthermore, GPS was used to record latitude and longitude in each elevation zone, while a clinometer was used to measure aspect angle.

### 2.4 Statistical analyses

Circular analysis was used to evaluate the phenological sequence in the yearly life cycle by using the Oriana version 2.0 packages. A 52‐week circular diagram was constructed expressing the beginning and end of each phenophase, lasting from January–December. Linear regression model and correlation function was used to estimate the relationship between climatic condition and phenophases across the habitats invaded by *X. strumarium*. Descriptive statistics and ANOVA were used to evaluate the inter‐altitude group variation in PFTs. PFTs and elevation relationship was evaluated by the general linear model and correlation functions to estimate the predictor strength. Tukey's honest significance test for multiple comparisons of group means was used to determine significant effects, with significance set at *p* ≤ .05. All data were analyzed in SPSS version 22, and graphical representation was generated in Sigma plot version 14.

## 3 RESULTS

### 3.1 Phenology and life‐cycle of *X. strumarium*

**FIGURE 2:**
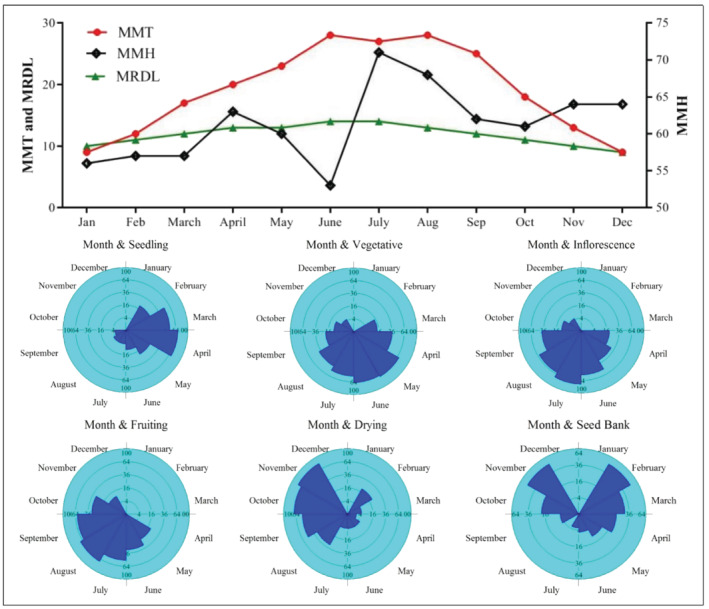
Climatic diagram showing mean monthly temperature (MMT, °C), humidity (MMH, %) and relative day length (MRDL, hours) with a circular histogram for phenological stages of *Xanthium strumarium* across the elevation gradient

Growing season duration is an essential parameter in the plant life cycle and phenological phase appearance and establishment of a species in the introduced region. Variation in these stages is mainly dependent on seasonal differences (Spring‐Autumn) and climatic factors. The phenological diagram for *X*. *strumarium* is summarized in Figure 2. Vegetative and reproductive development occurs mainly in the summer season, mostly matching higher temperature and humidity months. In general, the phenophases of vegetative and reproductive life cycle overlap across the elevation gradient starting from March and were visible until September, peaking in June–August. The inflorescence is generally initiated in summer (May–June) and almost finished in late summer (July), crowning in June. The fruit formation started in June and was sustained until August, and climaxing in late July.

Fruits dehiscence reached its peak in August–September and in the record to culminate in October, though, its occurrence remained until November in dried plants. The incidence of the vegetative and reproductive phenophases was found as well in the populations under consideration across the altitudinal gradient; but, it was necessary to note the alterations in the starting and finishing/ending period of the phenophases among populations (Figure 2). In general, phenophases in the lower altitudinal populations began and ended earlier than at higher elevations. In lower altitudinal areas, the beginning of April is the start of a vegetative phenophase lasting for a month, finishing late in the month of May. The inflorescence phenophase began in early June at the lower altitude, whereas the other populations began approximately a month later. In comparison, it began later in the higher altitudinal population and ending in mid‐June. The advent of fruit formation at the lower altitude appeared earlier and, interestingly, in all populations, the peak occurred a month before between low altitude and higher altitude populations.

Step wise regression showed the influence of climatic conditions on the appearance of *X*. *strumarium* vegetative and reproductive phenophases and was found well felt in all months before each phenophase emergence (Table 1). Seedlings development was shown to be directly affected by AMH (*R* ^2^ adj. = .37, F [5.106] = 37.499, *p* < .02). Relative to the seedling phenology, MMT, AMH and DLH of these 2 years were the most influential factors on vegetative growth (MMT: *R* ^2^ adj. = .23, *p* = .06; AMH: *R* ^2^ adj. = .74, *p* = .001; and DLH: *R* ^2^ adj. = .46, *p* < .01). In turn, the formation of reproductive phenophases (inflorescence) was determined by the MMT (*R* ^2^ adj. = .51, *P* < .01) and DLH (*R* ^2^ adj. = .49, *p* < .01). The MMT, AMH, and DLH for the year 2018–2019 were observed to be the main factors negatively influencing the D % and SB % phenology. The proportion of variations explained by MMT, AYH and DLH were 40%, 47%, and 65%, with D% and 81%, 27%, and 61% with SB%, respectively. F% was more related to MMT (having *R* ^2^ adj. = .59, *p* < .05).

**TABLE 1:** Regression model output and correlation between climatic condition and phenological stage of in *Xanthium strumarium* invaded areas of Khyber Pakhtunkhwa, Pakistan

Regression model		S %	V %	I %	F %	D %	SB %
	*R*	.41	.55*	.75**	.59*	−.67*	−.91**
X = MMT	*p* value	.83	.06	.005	.04	.01	.0001
	Ajd *R* ^2^	−.095	.23	.51	.28	.4	.81
	Equation	*ŷ* = −.13*X* + 14.50	*ŷ* = 1.33*X* − 1.00	*ŷ* = 1.5*X* − 17.61	*ŷ* = 2.31*X* − 2.92	*ŷ* = −1.49*X* + 43.45	*ŷ* = −3.54*X* + 90.76
X = AMH	*R*	.65**	.87**	.3	−.24	−.72**	−.53*
	*p* value	0.02	.0001	.33	.43	.008	.07
	Ajd *R* ^2^	.37	.74	.003	.03	.47	.21
	Equation	*ŷ* = 1.51*X* − 82.29	*ŷ* = 1.67*X* − 89.63	*ŷ* = .48*X* − 19.23	*ŷ* = −.77*X* + 71.96	*ŷ* = −1.26*X* + 94.27	*ŷ* = −1.65*X* + 126.63
X = DLH	*R*	.21	.71**	.73**	.29	−.82**	−.8**
	*p* value	.51	.009	.007	.35	.001	.001
	Ajd *R* ^2^	−.053	.46	.49	−.003	.65	.61
	Equation	*ŷ* = 2.63*X* − 19.69	*ŷ* = 7.43*X* − 73.86	*ŷ* = 6.31*X* − 64.84	*ŷ* = 4.97*X* − 36.45	*ŷ* = −7.83*X* + 109.11	*ŷ* = −13.4*X* + 184.9

*Note*: X (Climatic condition): *r* (Correlation): S% (Seedling percentage): V % (Vegetative phenology percentage): I % (Inflorescence phenology percentage): F % (Fruiting phenology percentage): D % (Drying phenology percentage): SB % (Seed bank phenology percentage).

* *p* < .05.

** *p* < .01.

### 3.2 Vegetative traits plasticity

**FIGURE 3:**
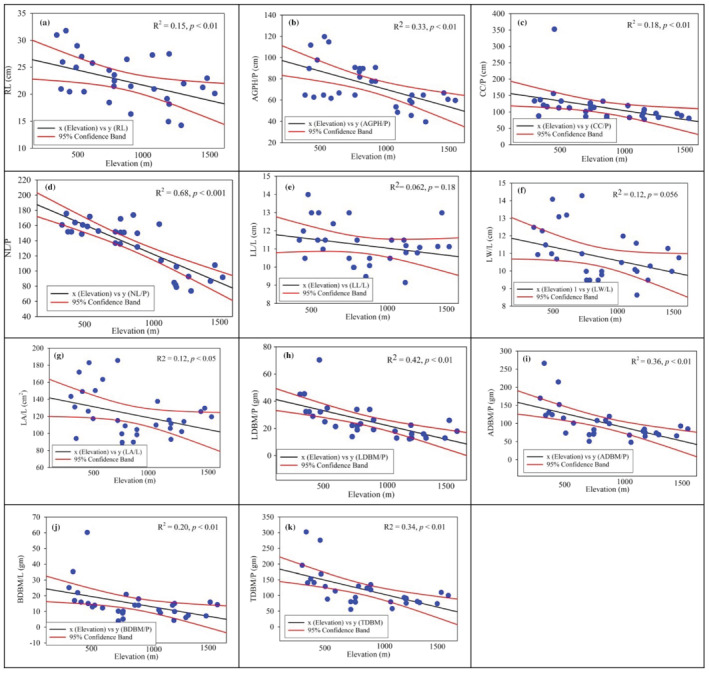
Pattern of non‐reproductive traits' variation in *Xanthium strumarium* along the altitudinal gradient (500, 1000, and 1500 m). (a) Root length per plant (RL/P; cm), (b) above ground plant height per plant (AGPH/P; cm), (c) crown cover per plant (CC/P; cm), (d) number of leaves per plant (NL/P), (e) leaf length per leaf (LL/L; cm), (f) leaf width per leaf (LW/L; cm), (g) leaf area per leaf (LA/L; cm^2^), (h) leaf dry biomass per plant (LDBM/P; g), (i) above‐ground dry biomass per plant (ADBM/P; g), (j) below‐ground dry biomass (BDBM/P; g), and (k) total dry biomass per plant (TDBM/P; g). *Note*: Blue circle represent the actual data points

The important vegetative phenotypic traits like AGPH/P (*R* ^2^ = .33, *p <* .01), NL/P (*R* ^2^ = .68, *p <* .01), LA/L (*R* ^2^ = .12, *p <* .05) and LDBM/P (*R* ^2^ = .42, *p <* .01) and biomass traits like BDBM/P (*R* ^2^ = .20, *p <* .05), AGDBM/P (*R* ^2^ = .36, *p <* .01) and TDBM (*R* ^2^ = .34, *p <* .01) exhibited negative relationship with altitude. On the other hand, the traits of LL/L (*R* ^2^ = .062, *p* = .18) presented a positive relationship across the altitudinal gradient **(**Figure 3 **)**. Linear regression model of vegetative traits plasticity displayed noteworthy differences in response to the altitudinal gradient in most of the traits such as RL/P (*t* = −2.52, *p* ≤ .01), AGPH/P (*t* = −3.78, *p* < .01), CC/P (*t* = −2.51, *p* ≤ .01), NL/P (*t* = −7.80, *p* < .001). Similarly, vegetative biomass variations, that is, LDBM/P (*t* = −4.51, *p ≪* .01), ADBM/P (*t* = −4.01, *p* < .01), BDBM/P (*t* = −2.64, *p* < .01), and TDBM/P (*t* = −3.87, *p* < .01) exposed noteworthy differences across the altitude **(**Table S2). Overall, the vegetative traits presented noteworthy correlation with altitude and soil characteristics (Table S3). The vegetative traits displayed highly negative correlation with respect to altitude where NL/P (*R* = −.83, *p <* .01), LDBM/P (*R* = −.61, *p* = .01), and ADBM (*R* = −.56, *p <* .01). Similarly, the climatic variable displays negative correlation along altitude, that is, MYT (*R* = −.92, *p* = .01), AYH (*R* = −.83, *p* = .01) and DLH (*R* = −.81, *p* = .01). In contrast soil variables show non‐significant correlation across the elevation gradient except OM % (*R* = −.61, *p* = .01). Likewise, in plant vegetative traits LL/L shows non‐significant correlation across the elevation gradient. All the vegetative traits showed the maximum mean value at 500 m and minimum at 1500‐m altitude (Table 2 and Figure S1).

**TABLE 2:** Variation of plant functional traits (PFT) (mean and standard error) of *Xanthium strumarium* along the elevational gradient (500 m, 1000 m and 1500 m. a.s.l.) in Pakistan

PFT	Elevation (m)			*F* value	*p* value
	500	1000	1500		
RL/P	25.75 ± 1.31^a^	21.47 ± 1.4^ab^	2.17 ± 1.21^c^	4.84	.016
AGPH/P	85.7 ± 7.5^a^	80.6 ± 3.9^a^	57.1 ± 2.8^b^	8.59	.001
CC/P	147.25 ± 23.61^a^	108.93 ± 5.69^ab^	91.07 ± 2.88^c^	4.13	.027
NL/P	159.9 ± 2.82^a^	151.4 ± 4.41^a^	92 ± 4.22^b^	90.62	1.05E−12
LL/L	12.04 ± .33^a^	10.46 ± .35^b^	11.136 ± .29^b^	5.82	.008
LW/L	11.83 ± .47^a^	1.30 ± .47^b^	1.42 ± .32^b^	3.91	.032
LA/L	143.31 ± 8.43^a^	109 ± 9.08^b^	115.88 ± 4.20^b^	5.76	.008
LDBM/P	37.05 ± 4.43^a^	23.43 ± 2.08^a^	16.48 ± 1.4^b^	12.56	.000
ADBM/P	148 ± 18.02^a^	86.17 ± 7.14^b^	74.94 ± 4.02^b^	11.87	.000
BDBM/P	23.22 ± 4.69^a^	11.87 ± 1.66^b^	10.61 ± 1.31^b^	5.45	.010
TDBM/P	171.25 ± 21.78^a^	98.04 ± 8.54^b^	85.55 ± 4.69^b^	11.29	.000
FDBM/P	32.94 ± 6.34^a^	19.5 ± 1.57^a^	22.3 ± 2.51^a^	3.06	.064
IDBM/P	8.36 ± .91^a^	7.4 ± .30^a^	4.91 ± .37^b^	9.04	.001
NC/P	242.88 ± 9.41^a^	216.88 ± 5.32^b^	145.11 ± 6.16^c^	49.64	9.02E−10
NF/P	562 ± 14.95^a^	486.66 ± 15.93^b^	280.15 ± 20.79^c^	7.21	2.00E−11
NC/NF	.43 ± 0.02^a^	.44 ± .01^a^	.47 ± 0.02^a^	.83	.45
IDBM/FDBM	.31 ± 04^a^	.40 ± .03^a^	.23 ± .02^b^	5.59	.01

*Note*: Different letter mean significant variations between the mean at *p* < .05.

### 3.3 Reproductive traits plasticity

**FIGURE 4:**
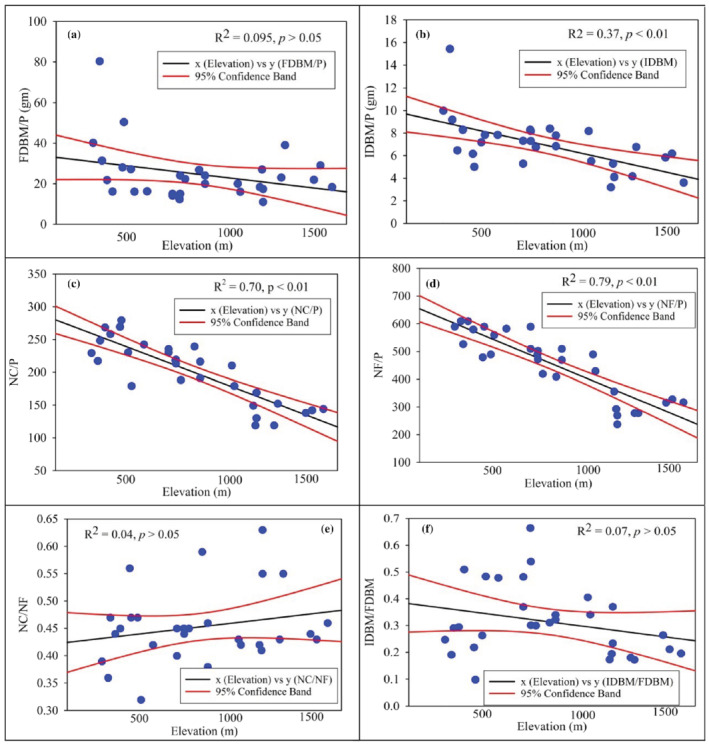
Pattern of the reproductive traits' variation in *Xanthium strumarium* along the altitudinal gradient. (a) Number of capitula per plant (NC/P), (b) number of fruits per plant (NF/P), (c) inflorescence dry biomass per plant (IDBM/P; g), (d) fruit dry biomass per plant (FDBM/P; g), (e) number of capitula to number of fruit (NC/NF), and (f) inflorescence dry biomass to fruit dry biomass (IDBM/FDBM). *Note*: Blue circle represent data points

**FIGURE 5:**
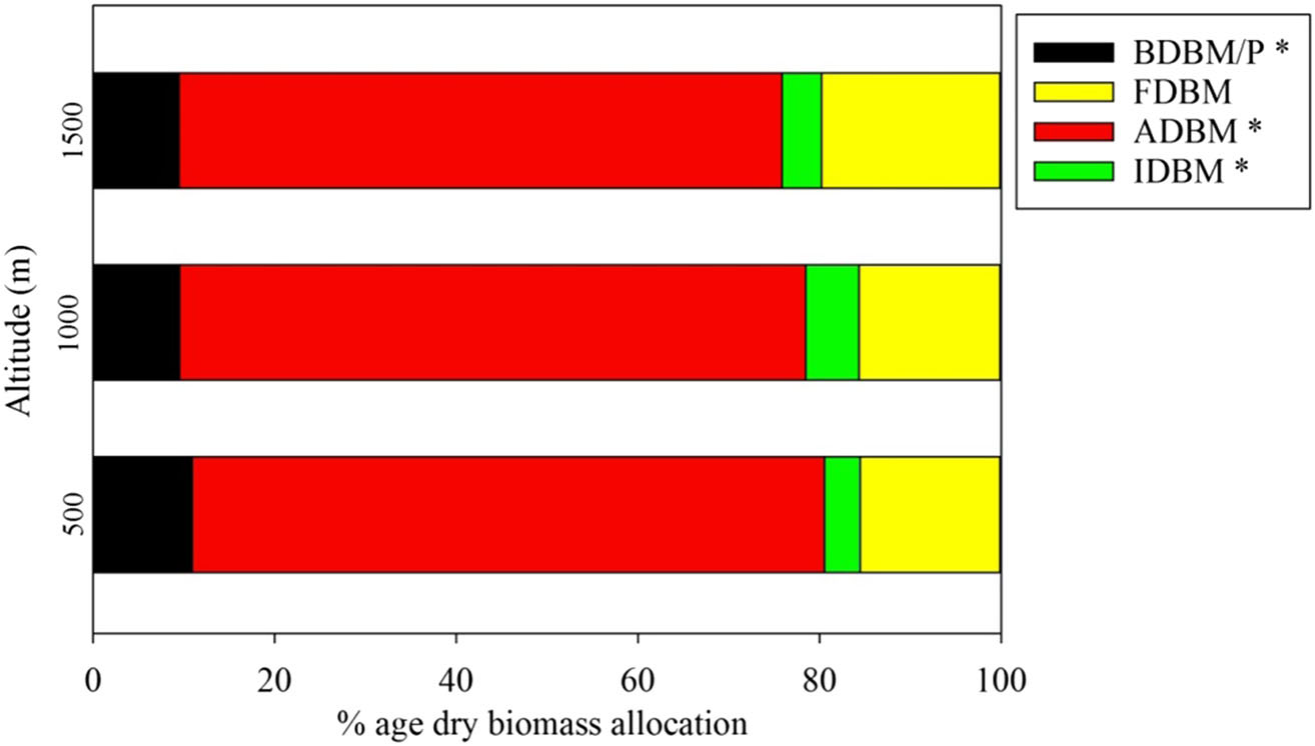
Dry biomass allocation between AGBM (above‐ground dry biomass/plant); BDBM/P (below‐ground dry biomass/plant); IDBM/P (inflorescence dry biomass/plant) and FDBM/P (fruit dry biomass/plant) of *Xanthium strumarium* at each elevation (500, 1000, and 1500 m). * denotes significance at *p <* .001

NC/P (*R* ^2^ = .70, *p* < .01), NF (*p* < .60, *p* < .001) and IDBM/P (*R* ^2^ = .37, *p* < .01) reduced continuously along the elevation gradient in reproductive traits, whereas FDBM/P (*R* ^2^ = .095, *p* > .05) had a significant linear relation across the elevation in reproductive traits (Figure 4). Invasive success was promising due to the non‐significant NC/NF linear regression (*R* ^2^ .04, *p* > .05) and IDBM/FDBM (*R* ^2^ = .07, *p* > .05). Linear regression models exposed significant disparities in the reproductive traits of *X. strumarium* such as NC/P (*t* = −8.23, *p <* .01), NF/P (*t* = −1.34, *p <* .01), IDBM/P (*t* = −4.07, *p <* .01) and FDBM/P (*t* = −1.72, *p <* .05) across the changing altitude (Table S2). The reproductive traits NF/P (R = −.87, *p <* .01) and NC/P (R = −.82, *p <* .01) displayed strong correlation with altitude, trailed by IDBM/P (*R* = −.57, *p <* .01) (Figure 4). Furthermore, reproductive trait ratios, that is, NC/NF and IDBM/FDBM were found non‐significantly correlated with latitudinal gradient (Table S3). In comparison, the OM % exhibited significant positive NF/P (*R* = .73, *p* < .01) and NC/P (*R* = .58, *p* < .01) correlations while the IDBM/P exhibited high relationship with N% (*R* = .49, *p* < .001) and FDBM/P (*R* = .38, *p* < .05) (Table S3). The minimum average value for NC/P, NF/P and FDBM/P were found at 1000 m, whereas IDBM showed the minimum average value at 1500 m (Table 2 and Figure S1). Allocation of biomass conveyed as percentage in above‐ground parts decreases with altitude (ADBM/P, *p <* .001) and BDBM (*p* < .01), while Fruit dry biomass (FDBM) shows non‐significant variation and increase across the elevation gradient (Figure 5).

### 3.4 Associated environmental variables

In soil characteristics, OM % (*F* = 11.48, *p* < .001), L % (*F* = 4.05, *p* < .05), EC (*F* = 3.81, *p* < .05), and TDS (*F* = 3.81, *p* < .05) presented significant correlations with elevation; however, soil texture and hydraulic properties varied non‐significantly with the increasing altitude (Table 3). Similarly, in geographic and climatic variables, such as N° (*F* = 2.99, *p* < .01), E° (*F* = 74.16, *p* < .01), MYT (*F* = 91.81, *p* < .01), AYH (*F* = 5.99, *p* < .01), and DLH (*F* = 38.33, *p* < .01) varies significantly across the elevation. Both soil and climate factors were correlated with changing altitude with R values ranges from −.36 to −.92. The L %, DLH, AYH, and AYT exhibited strong negative correlation while OM % revealed strong positive correlation, and N %, P (mg/kg), soil texture and hydraulic properties showed non‐significant correlation (Table 3). Soil nutrients such as OM % and L % and all the climatic variables were maximum at low elevation and minimum at higher elevation (Table 3).

**TABLE 3:** Variation of studied environmental parameters (mean and standard error) along the altitudinal gradient (500, 1000, and 1500 m a.s.l.)

Elevation	500	1000	1500	*F* value	*p* value
N°	34.1 ± .01a	34.61 ± .02b	34.35 ± .02c	20.996	3.16E−06
E°	71.85 ± .03a	72.31 ± .02b	73.17 ± .008c	74.166	1.07E−11
AA	159 ± 10	177.3 ± 8.3	143.4 ± 11.35	.2881	.752
Cl%	27.27 ± 1.2	29.99 ± 1.28	31.25 ± 1.2	.2692	.766
Si%	38.13 ± 1.87	33.32 ± 1.19	32.02 ± .8	.5563	.5798
Sa%	34.59 ± 1.5	36.68 ± 1.34	36.72 ± 1.64	.065	.9372
pH 1:5	6.91 ± .04	6.81 ± 0.02	6.88 ± .04	.1596	.8533
OM%	1.84 ± .06a	1.78 ± .06a	.72 ± .04b	11.486	.0002
L %	9.95 ± .12a	7.52 ± .17b	7.85 ± .22b	4.0555	.0288
N	.07 ± .005	.071 ± .004	.034 ± .003	1.8143	.1823
P	4.66 ± .09	4.97 ± .08	4.9 ± 0.1	.2747	.7619
K	106.9 ± 3.27	93.9 ± 2.2	103.6 ± 2.8	.5695	.5724
EC	244.9 ± 5.64a	337.1 ± 10.6b	332.8 ± 8.19b	3.8115	.0348
TDS	156.73 ± 3.61a	215.74 ± 6.83b	212.99 ± 5.24b	3.8115	.0348
WP	.16 ± .005	.17 ± .006	.18 ± .006	.3097	.7362
FC	.3 ± .006	.3 ± .006	.31 ± .007	.1057	.9001
D	1.36 ± .006	1.35 ± .009	1.34 ± .008	.0833	.9203
SP	.48 ± .002	.49 ± .003	.49 ± .003	.0812	.9222
AW	.14 ± .002	.13 ± .001	.13 ± .001	.6004	.5558
MYT	19.25 ± .05a	16.71 ± .08b	14.25 ± .09c	91.818	9.03E−13
AYH	64.88 ± .28a	54.97 ± .37b	47.23 ± .48c	50.991	6.78E−10
DLH	11.94 ± 0.02	11.47 ± 0.02	11.25 ± .003	38.339	1.29E−08

*Note*: Elev. (Elevation): N° (North): E° (East): Cl % (Clay percentage): Si% (Silt percentage): Sa% (Sand percentage): pH (Protenz Hydrogen): OM% (Organic Matter percentage): OC% (Organic carbon percentage): TC% (Total carbon percentage): L% (Lime percentage): N (Nitrogen percentage): P (Phosphorus in mg/kg): K (Potassium in mg/kg): EC (Electrical conductivity μs/cm): TDS (Total dissolve solid ppm 640 Scale): WP (Wilting Point at 1500 kPa): FC (Field capacity at 33 kPa): BD (Bulk Density in g/cm^3^): SP (Saturation at 0 kPa): AW (Available water): MYT (Mena yearly temperature in °C): AYH (Average yearly humidity in percentage): DLH (Day length in hours).

### 3.5 Environment and plasticity relationship

**FIGURE 6:**
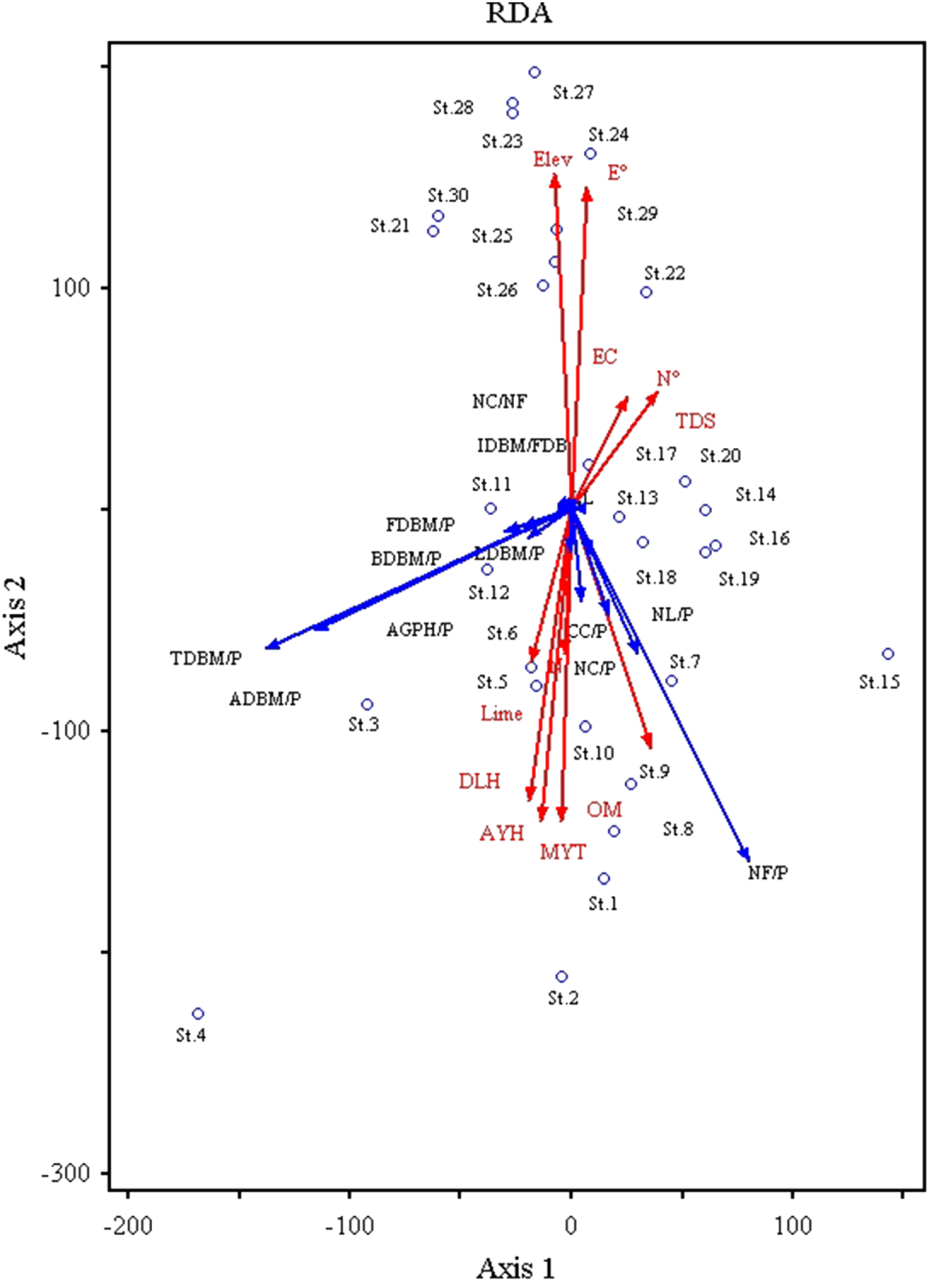
RDA bi‐plot for environmental variable affecting the plant functional traits of *Xanthium strumarium* along the altitudinal gradient

The RDA found that the major axis explained a significant proportion of characteristics and environment (85.9%). Possible combination i.e. Monte Carlo showed that the relationship between the characteristics and the environmental variables chosen by the model was meaningful. The first axis was defined negatively by some of the environmental variables such as elevation (*r* = −.92, *p* < .01), E° (*r* = −.88, *p* < .01) as depicted in Table 4. In contrast certain variables such as OM % (*r* = .66, *p* < .01), L % (*r* = .42, *p* < .05), N % (*r* = .40, *p* < .05), AYT (*r* = .86, *p* < .01), AYH (*r* = .86, *p* < .01), and DLH (*r* = .81, *p* < .01) exhibit strong positive correlation on axis 1. The RDA variables depicted in biplot revealing the significant factors affecting the biomass allocation across the elevation gradient (Figure 6).

**TABLE 4:** Redundancy analysis, correlation and bi‐plot scores of the associated environmental variables operated on *Xanthium strumarium* communities

Variable		Correlation			Biplot scores		
		Axis 1	Axis 2	Axis 3	Axis 1	Axis 2	Axis 3
1	Elev	−.92**	−.10	.06	−150.9	−7.84	3.50
2	N°	−.32	.50**	−.31	−52.31	38.92	−18.73
3	E°	−.88**	.09	−.11	−145.09	7.24	−6.89
4	AA	.06	−.12	.30	11.30	−9.70	17.98
5	Cl%	−.17	0.1	−.20	−29.09	7.68	−11.80
6	Si%	.27	−.09	.54**	44.31	−7.01	31.97
7	Sa%	−0.10	.001	−.33	−16.67	.11	−19.59
8	pH 1:5	.098	−.012	−.13	16.05	−.89	−8.09
9	OM%	.66**	.47**	.004	108.29	35.94	.24
10	L %	.42*	−.23	.01	69.53	−17.99	1.05
11	N	.40*	−.038*	.09	65.90	−2.93	5.42
12	P	−.21	0.11	.03	−35.11	8.93	1.74
13	K	−.00	.30	−.28	−.68	23.26	−16.80
14	EC	−.30	.33	−.02	−50.04	25.56	−1.3
15	TDS	−.30	.33	−0.02	−50.04	25.56	−1.33
16	WP	−.17	0.10	−.17	−28.97	8.26	−10.3
17	FC	−.09	.091	−.008	−15.12	6.96	−.44
18	D	0.10	−.01	.09	17.19	−0.99	5.55
19	SP	−0.10	.008	−.09	−17.1	.64	−5.63
20	AW	.29	−.149	.38*	47.89	−11.39	22.63
21	MYT	.86**	−.05	.006	141.01	−4.31	.33
22	AYH	.86**	−.17	.058	141.01	−13.58	3.38
23	DLH	.81**	−.2	.08	132.43	−19.13	4.72

* (*p* < .05).

** (*p* < .01).

## 4 DISCUSSIONS

This research shows that *X. strumarium* phenology differed across populations, and phenological activity lasted over the entire year. Vegetative and reproductive phenophases occurred in all populations in the following sequence: The emergence and development of phenophases occur from spring–summer and summer–autumn respectively and decrease entirely in winter to early spring. Instead, new leaves form throughout the growing season. The reproductive phenophase was on average identified as the most extended and lasted 4–6 months. This was substantially impacted by the duration of the fruit phenophase, which according to Anderson et al. (2005), one phenophase can affect subsequent phenophases. Overall, this phenological behavior is in line with that reported by Kaul (1971) for the same species in the Indian Kashmir seized in 1971. Of the earlier research in Indian occupied Kashmir, the significant variations in the timing of phenophases may be ascribed to the short period in the controlled situations, only masking the impact of interannual variability in phenology for one season (Dennill, 1987). The current research findings thus provide a more precise picture of the investigated species' phenological cycle.

The findings also showed significant variance in the populations phenological synchronization along altitudinal gradient, indicating that they did not develop at the same pace. The higher altitudes have the most significant synchronous index and the lowest altitudes. This result may be based on population size; the lower elevations of the other populations had more giant seedlings with higher plant density, which means they occupy a highly homogeneous environment. All plants were thus presumably tightly linked, and the synchronization may indicate genetic similarities (Buide et al., 2002). In turn, genetic variations are more likely to occur in a stressful environment (Goulart et al., 2005); presumably to explain why there is more considerable variability in high elevations across individuals.

The alien species will conserve their phenology in the invaded sites, and the climatic conditions will filter only those plant species whose phenology matches the favorable local conditions. In climatic filters, photoperiod is an essential factor that determined the phenological stages sequences and their maturation in the whole life cycle of a plant species. Kaul, in 1971 reported the phenological stage of *X. strumarium* in Indian occupied Kashmir and reported that critical photoperiod for different stages varies in range with a minimum of 13 hour continuous light. The finding of our study also reveals that photoperiod for different stages varies between 12 to 14 h; the difference may be attributed to many other regional, genetic, and environmental variables. Temperature and photoperiodic requirement of *X. strumarium* and various strains of *Lolium* have been studied by Cooper (1952), indicating the genus are of eastern Mediterranean or near eastern Asian origin as the strains from this region showed the lowest critical photoperiod. The results indicated that all the phenological stages of *X. strumarium* were strongly correlated with climatic variables, i.e., temperature, relative humidity, and day length. Such phenological variation in vegetative and reproductive growth phenologies were also studied by some authors, indicating that *Actaea spicata*, a member of the family Ranunculaceae, were dominated by temperature variations. Vegetative and reproductive stages changes were more likely to be determined by environmental characteristics in plots. Many other unexplained factors can also contribute to phenological variation other than the environmental/climatic factors, including differences in genetic makeup, small‐scale environmental heterogeneity, and history of the individual life cycle (Huang et al., 2016).

Earlier, the temperature was considered the sole dominant factor that determines flowering time and duration of many species (Diekmann, 1996; Scaven & Rafferty, 2013). The *X. strumarium* phenology was also found to be controlled by temperature factor; as the temperature rises, the vegetative and reproductively stages emerge and enhances while the decrease in temperature favors the dormant stages, that is, drying, seed production, and increase of seed bank. Changes in phenological attributes indicated that these stages and characters might also affect biotic factors like vegetation composition and abiotic‐like environmental changes. Climate change effect on phenology may be attributed to anthropogenic factors because of continuous human interruption in the natural ecosystem (Parry et al., 2007).

As observed from the strong positive regression among mean monthly temperature, mean monthly relative humidity, and photoperiod of daily length with various phenological stages, the changes in these environmental variables will shift the phenophases accordingly. Similarly, precipitation was the prominent factor in phenology, as reported by Parry et al. (2007). Plant species having more prolonged periods of vegetative and reproductive phenophases were exposed to sunlight for an extended duration resulting in a higher evapotranspiration rate which may affect the relative humidity and moisture content. A rise in gross and net primary productivity is also linked to the extension of the growing season, influencing crop production (Xia et al., 2015). Moreover, phenological variations influence seasonal animal migration, flora, matching, and even biological characteristics.

Inordinate diurnal fluctuation in climatic factors was reported at high altitudes compared to the lowland area (Pandey et al., 2006). The present study depicted clear and significant variations in vegetative and reproductive PFT's across the altitudinal gradient in *X. strumarium* except for the ratio of NC/NF. Many other studies on invasive plants also revealed a similar pattern of AGPH/P, RL/P, and seed mass variation was observed by Rathee et al. (2021) in *Parthenium hysterophorus* and dimension of the flower by Hattori et al. (2016) in member of family Balsaminaceae (*Impatiens textori* Miq.). Similarly, Yaqoob and Nawchoo (2017) reported the growth dynamic of *Ferula jaeschkeana* Vatke. and Datta et al. (2017) revealed the life history stages of *Ageratina adenophora* (Spreng.) R. M. King & H. Rob. The decrease in AGPH/P is the most significant change in vegetative characteristics, which may play a critical function in shielding plants from high wind speed and cold stress (Fabbro & Körner, 2004). The nearer the plant is to the earth's surface, the greater the build‐up of heat in the canopy of the leaves, which may have a beneficial impact on seed setting and, later on, safe dispersal (Fabbro & Körner, 2004). Height reduction also encourages pollinators to ensure high levels of reproductive fitness (Trunschke & Stöcklin, 2017). A reduction in BGB with altitude contradicted Yaqoob and Nawchoo's (2017) results. Area‐specific patterns may also be linked with altitudinal variation since various mountain ranges might have different relationships between altitude and environmental variables (Olejniczak et al., 2018).

Soil characteristics also adjust to altitude changes (Badía et al., 2016), e.g., with rising altitude, the OM %, L %, N % drop, while the TDS and EC increase. Soil chemical composition and nutrient availability regulated the invasion ability of a plant species either directly or indirectly (Sardans et al., 2017). Soil nutrition and invasive species ability to utilize available nutrients affect its succession or retrogression (Vasquez et al., 2008). Dassonville et al. (2008) found that foreign invasive plants may contribute to soil homogeneity and support further invasions in occupied environments. Invasive plants are often related to higher pools of C, N, P, and K (Sardans et al., 2017). Cowie et al. (2019) have shown an increase in the growth rate and biomass production of the *X. strumarium* in nutrient‐rich environments (alongside plant biomass variations). Furthermore, environmental degradation and perturbance promote invaders such as *P. hysterophorus* (Rathee et al., 2021; Seta et al., 2013) and *X. strumarium* (Ullah et al., 2021). A combination of soil instability and fertilizer addition was found to have the most significant effect on the promotion and development of non‐native species (Hobbs & Huenneke, 1992). Studies have revealed that soil nutrient and plant invasion have a significant relationship (Hester & Hobbs, 1992). Osunkoya et al. (2017) reported enhanced decomposition and microbial activities in soils invaded by *P*. *hysterophorus*. The same has been proven true for *X*. *strumarium* in the present scenario when nutrient‐rich areas are mainly invaded. In our research, we recorded that reduced nutrient availability rat altitudes over 1000 m may reduce biomass allocation to above‐ground parts, particularly vegetative parts, and decrease the allocation of biomass to below‐ground parts.

Temperature shifts would ultimately threaten most indigenous species, while alien species that are easily established in warm regions would flourish (Hou et al., 2014). In addition, plant invasion and development are also correlated to other global variables such as rainfall, Nitrogen and Carbon Dioxide deposition (Hou et al., 2014). Many authors have argued that increasing precipitation also produces nutrient‐richness and enhances intrusions (Eskelinen & Harrison, 2014). Our research reported that *X. strumarium* adapted to a broad range of elevation‐wide environmental gradients. Its effective growth and propagation showed its adaptability with phenotypic plasticity in various climatic and soil conditions. Similar phenotypic plasticity and broad adaption range were observed in *P. hysterophorus* by many researchers, for example, Datta et al., 2017 showed its adaptability to various environmental gradients. In addition, Annapurna and Singh (2003) and Kadam et al. (2009) reported that the species exhibited plasticity in varied soil types and air contamination pressure. Nguyen et al. (2017) have shown that invasive weeds such as *P. hysterophorus* are well‐adapted to changes in climatic factors like CO_2_, water scarcity and elevated temperature. In natural environments, the biomass allocation in plants shows different patterns, focusing on allometric trajectories and others are showing significant resource allocation flexibility (Weiner, 2004). The allocation of resources, reproduction and stress tolerance were probable major selection forces for adapting species fitness (Fabbro & Körner, 2004). Redmond et al. (2019) showed that allocation of resources and its trade‐off in different types of biotic and abiotic stresses varies among populations/individuals.

Our results show that nutrient rich soils at lower altitudes have enhanced vegetative and reproductive output of *X. strumarium* since plants do not need more of their energy to acquire these nutrients (Berntson & Wayne, 2000). Our findings were also similar to Datta et al. (2017), who revealed higher plant biomass at lower altitudes in *A. adenophora*. This study contradicts Zhigang et al. (2006), who stated that the high terrestrial nutrient status of the higher altitudes could be responsible for the observed trend that biomass allocation not reduced at altitude in *Anemone rivularis* Buch.–Ham ex DC. (Ranunculaceae), *A. obtusiloba* D. Don (Ranunculaceae). Invasive species allocate higher reproductive biomass in stress conditions like temperature, light intensity, and nutrient absorption along the altitudinal gradient than vegetative parts to complete a life cycle (Wenk & Falster, 2015). Similarly, *X. strumarium* maintained reproductive output at higher elevations in mountain environments as an adaptive strategy as educated by Arroyo et al. (2013). Consequently, enhancing reproductive fitness prioritizes vegetative fitness in *X. strumarium* along elevation. Besides MYT, AYH, DLH, and altitude, additional environmental variables like soil OM %, N %, EC, TDS, N, and E may be significant in PFT's plasticity and are described by authors such as Cheng et al. (2015), Mason et al. (2017), and Xue et al. (2018). In addition, genetic differentiation, gene flow, and phenotypic plasticity may provide plants with an adaptive ability to dealing with climatic change along altitude gradients (Gonzalo‐Turpin & Hazard, 2009). Recent research found that specific genes related to local temperature and abiotic stress response affected growth allometry in *Arabidopsis thaliana* genomic analysis (Vasseur et al., 2018). The species, however, exhibited distinct biomass allocation methods throughout the altitude gradient. However, vegetative and reproductive biomass and phenotypic plasticity of *X. strumarium* were substantially associated with elevation while positively correlated with OM%, N%, L%, and climatic factors. Similarly, Zhang et al. (2020) found a similar pattern of environmental factors influencing the allocation of biomass and plasticity of two *Gentiana* species along the altitude gradient in Yunnan‐Guizhou Plateau, China. Further researches are required to explore additional environmental variables and genetic components, to understand vegetative and reproductive plasticity strategies throughout the environmental gradients.

## 5 CONCLUSIONS

Formerly, high altitude sites were considered unsuitable in comparison to lowland regions for invasion, but the process is now accelerated by human disturbances (direct or indirect) and climate change. Studies show that mountain ecosystems are no longer resistant to invasion and are of environmental and economic significance. It may be inferred from this study that *X. strumarium* can alter phenological and morphological features to expand its environmental and geographical range. The study has shown that reproductive features are essential to increase the adaptability of *X. strumarium*. These results provide a wide avenue for future study. It not only helps anticipate the behavior of *X. strumarium* but may also predict other invasive plant species such as those with phylogenetic or morphological similarities with *X. strumarium*. This study will be very crucial and useful broadening the understanding of the distribution, growth and expansion of alien invasive species along different environmental gradient in the context of observed and anticipated rapid climate change.

## CONFLICT OF INTEREST

The Authors did not report any conflict of interest.

## Supporting information


**Fig. S1** Whisker‐plots for plants functional traits variation across the altitudinal groups
**Table S2** General linear Model details for different phenotypic plasticity relation across the elevation gradient as predictor variable
**Table S3.** Correlation function among the various environmental and plant functional traits parameters
