# Supplementary material for: Which factor explains the life‐history of Xanthium strumarium L., an aggressive alien invasive plant species, along its altitudinal gradient?
Source: Plant Direct. 2022 Jan 9;6(1):e375. doi: 10.1002/pld3.375 (PMC8743363; doi:10.1002/pld3.375)
Supplement: Supplementary file 1 — Fig. S1 Whisker‐plots for plants functional traits variation across the altitudinal groups Table S2 General linear Model details for different phenotypic plasticity relation across the elevation gradient as predictor variable Table S3. Correlation function among the various environmental and plant functional traits parameters [file PLD3-6-e375-s001.docx]

| 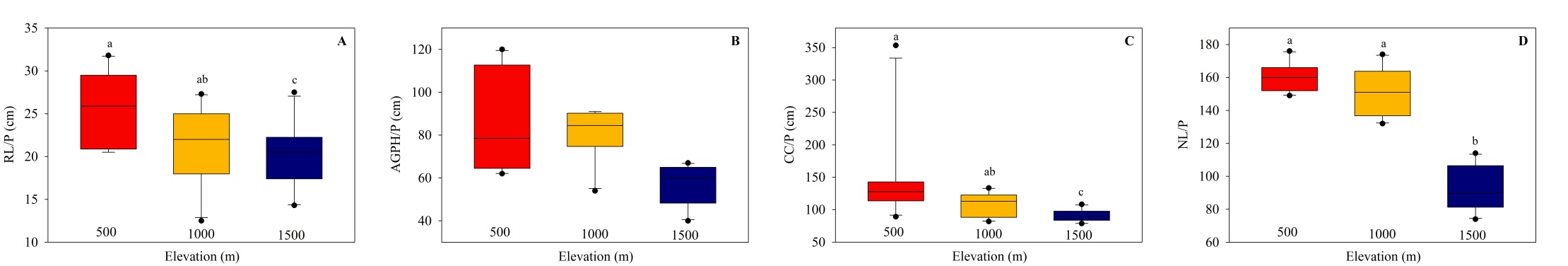 |
| --- |
| 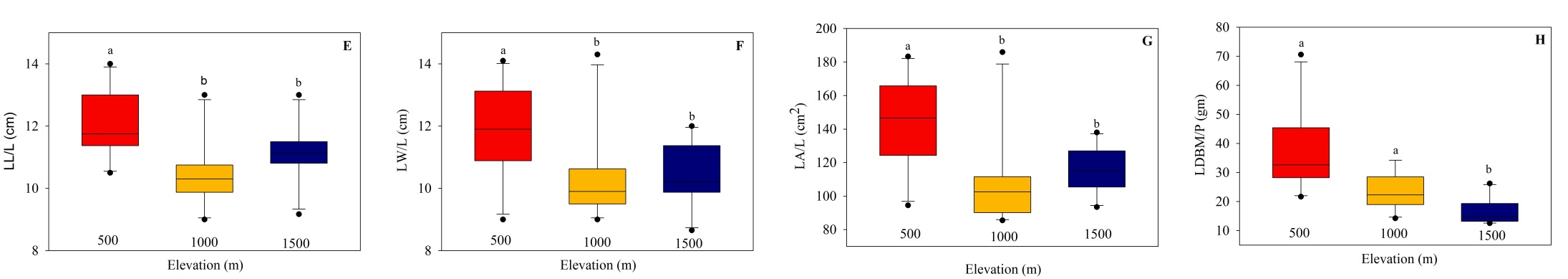 |
| 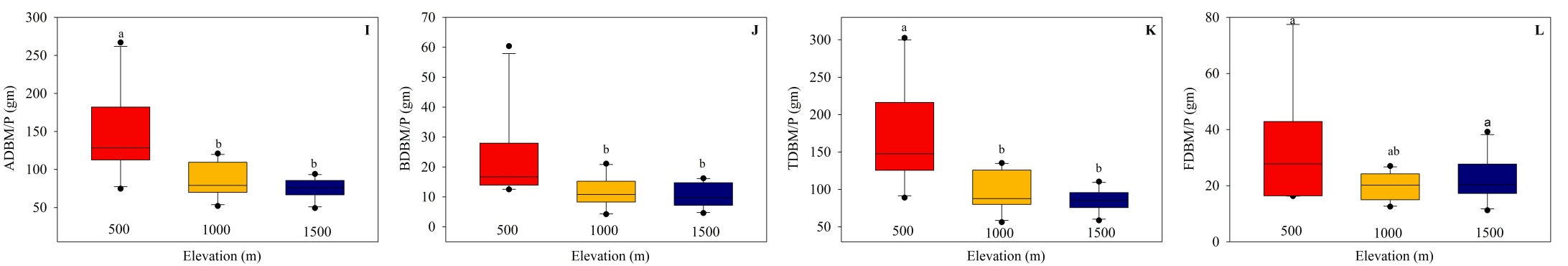 |
| 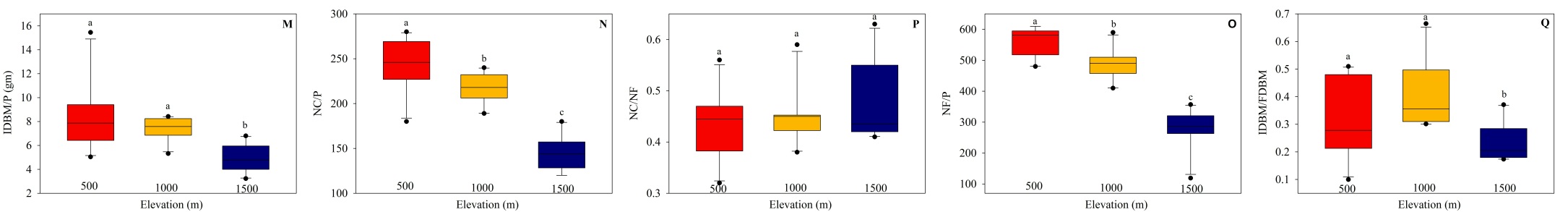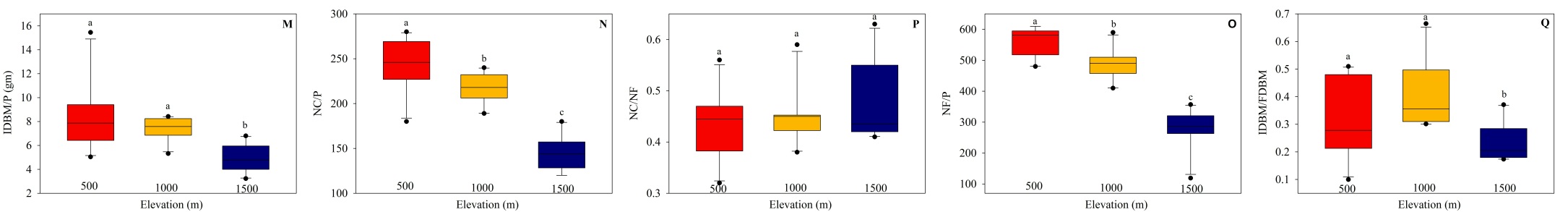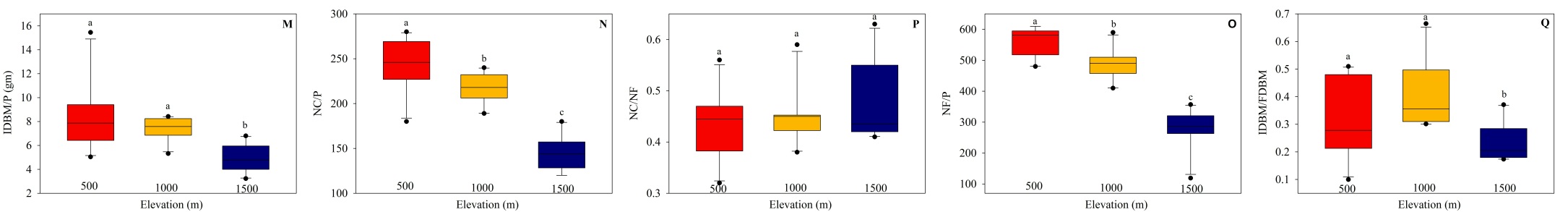 |
| 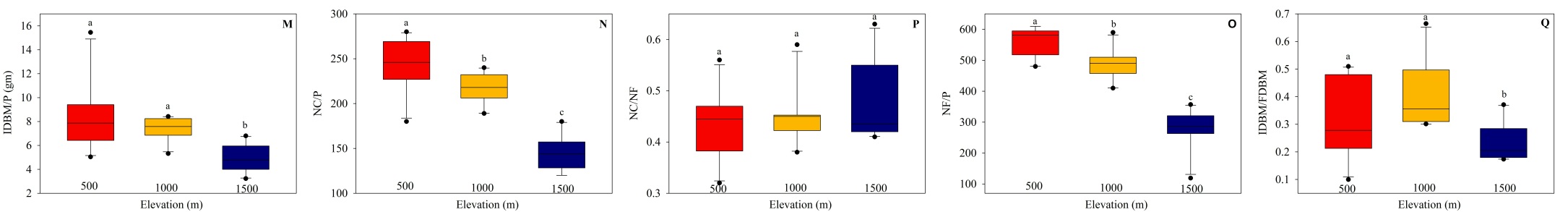 |

**Fig. S1** Whisker-plots for plants functional traits variation across the altitudinal groups

*Different letters shows significant at p < 0.05 by Tukey HSD

**Table S2** General linear Model details for different phenotypic plasticity relation across the elevation gradient as predictor variable

| R(V) | Estimate | SE | t-value | *p*-value | DF | F-value | PC (r) |
| --- | --- | --- | --- | --- | --- | --- | --- |
| RL/P | 0.005 | 0.002 | -2.52 | ≤ 0.01 | 1, 28 | 6.3^**^ | -0.43^**^ |
| AGPH/P | -0.029 | 0.007 | -3.78 | <0.01 | 1, 28 | 14.3^**^ | -0.58^**^ |
| CC/P | -0.05 | 0.02 | -2.51 | ≤ 0.01 | 1, 28 | 6.3^**^ | -0.42^**^ |
| NL/P | -0.068 | 0.008 | -7.80 | < 0.001 | 1, 28 | 60.9^**^ | -0.82^**^ |
| LL/L | -0.0008 | 0.0006 | -1.36 | >0.05 | 1, 28 | 1.58 | -0.24 |
| LW/L | -0.0013 | 0.0007 | -1.99 | = 0.05 | 1, 28 | 3.97^*^ | -0.35^*^ |
| LA/L | -0.024 | 0.012 | -2.04 | <0.05 | 1, 28 | 4.17^*^ | -0.36^*^ |
| LDBM/P | -0.020 | 0.004 | -4.51 | <0.01 | 1, 28 | 20.42^**^ | -0.64^**^ |
| ADBM/P | -0.072 | 0.01 | -4.01 | <0.01 | 1, 28 | 16.10^**^ | -0.60^**^ |
| BDBM/P | -0.01 | 0.004 | -2.64 | < 0.01 | 1, 28 | 7.0^**^ | -0.44^**^ |
| TDBM/P | -0.08 | 0.02 | -3.87 | <0.01 | 1, 28 | 15.04^**^ | -0.59^**^ |
| FDBM/P | -0.01 | 0.006 | -1.72 | >0.05 | 1, 28 | 2.96 | -0.30 |
| IDBM/P | -0.003 | 0.0009 | -4.07 | >0.001 | 1, 28 | 16.0^**^ | -0.61^**^ |
| NC/P | -0.094 | 0.011 | -8.23 | >0.001 | 1, 28 | 67.8^**^ | -0.84^**^ |
| NF/P | -0.21 | 0.021 | -10.34 | >0.001 | 1, 28 | 107.1^**^ | -0.89^**^ |
| NC/NF | 3.6E-005 | 3.06E-005 | 1.19 | > 0.05 | 1, 28 | 1.43 | 0.22 |
| IDBM/FDBM | -8.6E-005 | 5.9E-005 | -1.45 | >0.05 | 1, 28 | 2.10 | -0.26 |

**Table S3.** Correlation function among the various environmental and plant functional traits parameters

|  | Elev | N° | E° | AA | Cl% | Si% | Sa% | pH1:5 | OM% | L % | N | P | K | EC | TDS | WP | FC | D | SP | AW |
| --- | --- | --- | --- | --- | --- | --- | --- | --- | --- | --- | --- | --- | --- | --- | --- | --- | --- | --- | --- | --- |
| Elev | 1 |  |  |  |  |  |  |  |  |  |  |  |  |  |  |  |  |  |  |  |
| N° | .345 | 1 | .328 |  |  |  |  |  |  |  |  |  |  |  |  |  |  |  |  |  |
| E° | .87^**^ | .328 | 1 |  |  |  |  |  |  |  |  |  |  |  |  |  |  |  |  |  |
| AA | -.137 | -.188 | -.13 | 1 |  |  |  |  |  |  |  |  |  |  |  |  |  |  |  |  |
| Cl% | .113 | .156 | .197 | -.13 | 1 |  |  |  |  |  |  |  |  |  |  |  |  |  |  |  |
| Si% | -.140 | -.160 | -.26 | -.09 | -.343 | 1 |  |  |  |  |  |  |  |  |  |  |  |  |  |  |
| Sa% | .035 | .019 | .082 | .198 | -.50^**^ | -.6^**^ | 1 |  |  |  |  |  |  |  |  |  |  |  |  |  |
| pH1:5 | -.055 | .041 | .065 | -.15 | .150 | -.05 | -.07 | 1 |  |  |  |  |  |  |  |  |  |  |  |  |
| OM% | -.61^**^ | .008 | -.5^**^ | .033 | -.047 | .201 | -.14 | .178 | 1 |  |  |  |  |  |  |  |  |  |  |  |
| L % | -.36^*^ | -.36^*^ | -.23 | .077 | .004 | .320 | -.29 | .200 | .237 | 1 |  |  |  |  |  |  |  |  |  |  |
| N | -.290 | -.102 | -.38^*^ | .028 | -.184 | .197 | -.03 | -.11 | .247 | .031 | 1 |  |  |  |  |  |  |  |  |  |
| P | .089 | .045 | .058 | .240 | -.064 | -.28 | .310 | -.18 | -.12 | -.27 | .049 | 1 |  |  |  |  |  |  |  |  |
| K | -.060 | .072 | .141 | -.14 | .152 | -.35 | .205 | .020 | -.02 | -.02 | -.36^*^ | .377^*^ | 1 |  |  |  |  |  |  |  |
| EC | .348 | .411^*^ | .327 | -.08 | .105 | -.09 | .005 | .068 | .000 | -.35 | -.167 | .309 | .215 | 1 |  |  |  |  |  |  |
| TDS | .348 | .411^*^ | .327 | -.08 | .105 | -.09 | .005 | .068 | .000 | -.35 | -.167 | .309 | .215 | 1.^**^ | 1 |  |  |  |  |  |
| WP | .120 | .148 | .213 | -.09 | .996^**^ | -.35 | -.4^**^ | .153 | -.06 | .005 | -.172 | -.061 | .153 | .092 | .092 | 1 |  |  |  |  |
| FC | .074 | .107 | .131 | -.14 | .945^**^ | -.02 | -.7^**^ | .141 | .012 | .118 | -.119 | -.163 | .038 | .062 | .062 | .94^**^ | 1 |  |  |  |
| D | -.079 | -.110 | -.11 | .228 | -.92^**^ | .018 | .74^**^ | -.15 | -.01 | -.10 | .130 | .159 | -.045 | -.118 | -.118 | -.90^**^ | -.9^**^ | 1 |  |  |
| SP | .078 | .108 | .110 | -.27 | .929^**^ | -.01 | -.7^**^ | .154 | .018 | .110 | -.130 | -.157 | .042 | .118 | .118 | .90^**^ | .96^**^ | -1.0^**^ | 1 |  |
| AW | -.160 | -.131 | -.24 | -.14 | .032 | .90^**^ | -.8^**^ | .029 | .197 | .40^*^ | .219 | -.394^*^ | -.362^*^ | -.122 | -.122 | .014 | .339 | -.351 | .352 | 1 |
| MYT | -.92^**^ | -.43^*^ | -.8^**^ | .122 | -.024 | .095 | -.06 | -.03 | .50^**^ | .40^*^ | .259 | -.026 | .010 | -.386^*^ | -.386^*^ | -.033 | .001 | -.004 | .007 | .143 |
| AYH | -.82^**^ | -.53^**^ | -.8^**^ | .152 | -.126 | .149 | -.03 | .016 | .57^**^ | .37^*^ | .239 | -.108 | -.028 | -.246 | -.246 | -.140 | -.09 | .068 | -.06 | .159 |
| DLH | -.79^**^ | -.55^**^ | -.8^**^ | .115 | -.109 | .167 | -.06 | .192 | .47^**^ | .188 | .180 | -.175 | -.047 | -.284 | -.284 | -.119 | -.06 | .038 | -.03 | .182 |
| RL/P | -.45^*^ | -.266 | -.4^*^ | -.10 | -.196 | .157 | .018 | -.30 | .273 | .023 | .091 | -.149 | .105 | -.234 | -.234 | -.172 | -.13 | .200 | -.20 | .081 |
| AGPH/P | -.55^**^ | -.144 | -.6^**^ | .204 | -.009 | .113 | -.09 | .018 | .331 | .135 | .347 | .007 | .075 | -.156 | -.156 | -.018 | .012 | -.042 | .042 | .114 |
| CC/P | -.44^*^ | -.42^*^ | -.5^**^ | .273 | -.267 | .60^**^ | -.33 | -.04 | .350 | .208 | .296 | -.102 | -.264 | -.160 | -.160 | -.245 | -.05 | .141 | -.14 | .48^**^ |
| NL/P | -.85^**^ | -.111 | -.7^**^ | .160 | -.166 | .156 | -.00 | .001 | .62^**^ | .288 | .332 | -.033 | -.031 | -.262 | -.262 | -.173 | -.12 | .137 | -.13 | .164 |
| LL/L | -.23 | -.40^*^ | -.17 | -.20 | -.178 | .218 | -.05 | .296 | .348 | .56^**^ | .016 | -.271 | .232 | -.172 | -.172 | -.165 | -.10 | .118 | -.12 | .176 |
| LW/L | -.37^*^ | -.210 | -.30 | -.02 | -.123 | .243 | -.12 | .056 | .59^**^ | .303 | .193 | -.094 | .069 | .022 | .022 | -.111 | -.03 | .076 | -.07 | .213 |
| LA/L | -.36^*^ | -.312 | -.29 | -.11 | -.165 | .267 | -.10 | .178 | .56^**^ | .45^*^ | .155 | -.183 | .155 | -.062 | -.062 | -.151 | -.06 | .106 | -.10 | .224 |
| LDBM/P | -.60^**^ | -.48^**^ | -.7^**^ | .079 | -.112 | .184 | -.07 | .010 | .277 | .266 | .083 | -.127 | -.200 | -.288 | -.288 | -.133 | -.07 | .023 | -.01 | .217 |
| ADBM/P | -.54^**^ | -.53^**^ | -.6^**^ | .120 | -.169 | .211 | -.05 | .085 | .134 | .44^*^ | .312 | -.227 | -.197 | -.427^*^ | -.427^*^ | -.173 | -.11 | .070 | -.06 | .275 |
| BDBM/P | -.40^*^ | -.42^*^ | -.5^**^ | .011 | -.193 | .209 | -.03 | .041 | .010 | .327 | .035 | -.110 | -.082 | -.310 | -.310 | -.207 | -.14 | .096 | -.09 | .220 |
| TDBM/P | -.53^**^ | -.53^**^ | -.6^**^ | .103 | -.178 | .217 | -.05 | .079 | .115 | .43^*^ | .269 | -.211 | -.181 | -.417^*^ | -.417^*^ | -.184 | -.12 | .077 | -.07 | .272 |
| FDBM/P | -.262 | -.48^**^ | -.33 | .293 | -.169 | .257 | -.09 | .027 | .074 | .335 | .386^*^ | -.420^*^ | -.315 | -.381^*^ | -.381^*^ | -.154 | -.08 | .074 | -.07 | .340 |
| IDBM/P | -.61^**^ | -.208 | -.5^**^ | .119 | -.088 | -.11 | .181 | -.05 | .356 | .186 | .493^**^ | -.230 | -.125 | -.332 | -.332 | -.094 | -.14 | .106 | -.10 | -.009 |
| NC/P | -.83^**^ | -.114 | -.8^**^ | .119 | -.101 | .218 | -.11 | -.02 | .58^**^ | .332 | .227 | -.044 | .116 | -.232 | -.232 | -.094 | -.02 | .066 | -.06 | .206 |
| NF/P | -.89^**^ | -.169 | -.7^**^ | -.01 | -.127 | .184 | -.06 | .126 | .73^**^ | .341 | .384^*^ | -.209 | .084 | -.205 | -.205 | -.127 | -.06 | .085 | -.08 | .213 |
| NC/NF | .252 | -.024 | .202 | .108 | .243 | .001 | -.20 | -.34 | -.39^*^ | -.18 | -.083 | .266 | .200 | .017 | .017 | .267 | .28 | -.223 | .220 | .049 |
| IDBM/FDBM | -.314 | .353 | -.21 | -.18 | .027 | -.25 | .212 | -.07 | .333 | -.16 | .202 | .239 | .147 | .209 | .209 | .013 | -.06 | .057 | -.05 | -.271 |
|  | MYT | AYH | DLH | RL/P | AGPH/P | CC/P | NL/P | LL/L | LW/L | LA/L | LDBM/P | ADBM/P | BDBM/P | TDBM/P | FDBM/P | IDBM/P | NC/P | NF/P | NC/NF | IDBM/FDBM |
| MYT | 1 |  |  |  |  |  |  |  |  |  |  |  |  |  |  |  |  |  |  |  |
| AYH | .81^**^ | 1 |  |  |  |  |  |  |  |  |  |  |  |  |  |  |  |  |  |  |
| DLH | .77^**^ | .88^**^ | 1 |  |  |  |  |  |  |  |  |  |  |  |  |  |  |  |  |  |
| RL/P | .462^*^ | .44^*^ | .40^*^ | 1 |  |  |  |  |  |  |  |  |  |  |  |  |  |  |  |  |
| AGPH/P | .56^**^ | .53^**^ | .50^**^ | .102 | 1 |  |  |  |  |  |  |  |  |  |  |  |  |  |  |  |
| CC/P | .426^*^ | .46^**^ | .47^**^ | .38^*^ | .495^**^ | 1 |  |  |  |  |  |  |  |  |  |  |  |  |  |  |
| NL/P | .82^**^ | .75^**^ | .62^**^ | .44^*^ | .556^**^ | .37^*^ | 1 |  |  |  |  |  |  |  |  |  |  |  |  |  |
| LL/L | .180 | .399^*^ | .327 | .241 | .087 | .248 | .100 | 1 |  |  |  |  |  |  |  |  |  |  |  |  |
| LW/L | .301 | .389^*^ | .331 | .257 | .023 | .40^*^ | .219 | .60^**^ | 1 |  |  |  |  |  |  |  |  |  |  |  |
| LA/L | .289 | .446^*^ | .37^*^ | .291 | .085 | .39^*^ | .206 | .86^**^ | .92^**^ | 1 |  |  |  |  |  |  |  |  |  |  |
| LDBM/P | .65^**^ | .74^**^ | .72^**^ | .330 | .235 | .37^*^ | .54^**^ | .142 | .150 | .158 | 1 |  |  |  |  |  |  |  |  |  |
| ADBM/P | .61^**^ | .68^**^ | .69^**^ | .277 | .344 | .309 | .47^**^ | .269 | .126 | .204 | .812^**^ | 1 |  |  |  |  |  |  |  |  |
| BDBM/P | .402^*^ | .57^**^ | .55^**^ | .255 | .075 | .174 | .338 | .253 | .036 | .132 | .844^**^ | .825^**^ | 1 |  |  |  |  |  |  |  |
| TDBM/P | .59^**^ | .67^**^ | .69^**^ | .281 | .303 | .292 | .45^*^ | .273 | .112 | .196 | .841^**^ | .994^**^ | .881^**^ | 1 |  |  |  |  |  |  |
| FDBM/P | .314 | .411^*^ | .45^*^ | .198 | .235 | .310 | .243 | .186 | .123 | .162 | .438^*^ | .766^**^ | .417^*^ | .721^**^ | 1 |  |  |  |  |  |
| IDBM/P | .58^**^ | .58^**^ | .47^**^ | .203 | .349 | -.00 | .66^**^ | .007 | .069 | .051 | .475^**^ | .594^**^ | .310 | .556^**^ | .594^**^ | 1 |  |  |  |  |
| NC/P | .77^**^ | .72^**^ | .61^**^ | .59^**^ | .583^**^ | .52^**^ | .80^**^ | .304 | .338 | .38^*^ | .54^**^ | .488^**^ | .423^*^ | .489^**^ | .178 | .406^*^ | 1 |  |  |  |
| NF/P | .78^**^ | .75^**^ | .70^**^ | .45^*^ | .480^**^ | .38^*^ | .79^**^ | .40^*^ | .53^**^ | .55^**^ | .460^*^ | .466^**^ | .271 | .442^*^ | .293 | .62^**^ | .81^**^ | 1 |  |  |
| NC/NF | -.214 | -.250 | -.24 | .258 | -.082 | .067 | -.22 | -.18 | -.40^*^ | -.33 | -.019 | -.065 | .145 | -.027 | -.124 | -.338 | .019 | -.373^*^ | 1 |  |
| IDBM/FDBM | .185 | .092 | -.06 | -.06 | .154 | -.16 | .342 | -.13 | .066 | -.00 | -.054 | -.329 | -.248 | -.322 | -.55^**^ | .254 | .228 | .348 | -.20 | 1 |
| **. Correlation is significant at the 0.01 level (2-tailed). | | | | | | | | *. Correlation is significant at the 0.05 level (2-tailed). | | | | | | | | | | | |  |
